# Functional Analysis of the Na^+^,K^+^/H^+^ Antiporter PeNHX3 from the Tree Halophyte *Populus euphratica* in Yeast by Model-Guided Mutagenesis

**DOI:** 10.1371/journal.pone.0104147

**Published:** 2014-08-05

**Authors:** Liguang Wang, Xueying Feng, Hong Zhao, Lidong Wang, Lizhe An, Quan-Sheng Qiu

**Affiliations:** MOE Key Laboratory of Cell Activities and Stress Adaptations, School of Life Sciences, Lanzhou University, Lanzhou, Gansu, China; University of Cambridge, United Kingdom

## Abstract

Na^+^,K^+^/H^+^ antiporters are H^+^-coupled cotransporters that are crucial for cellular homeostasis. *Populus euphratica*, a well-known tree halophyte, contains six Na^+^/H^+^ antiporter genes (*PeNHX1-6*) that have been shown to function in salt tolerance. However, the catalytic mechanisms governing their ion transport remain largely unknown. Using the crystal structure of the Na^+^/H^+^ antiporter from the *Escherichia coli* (EcNhaA) as a template, we built the three-dimensional structure of PeNHX3 from *P. euphratica*. The PeNHX3 model displays the typical TM4-TM11 assembly that is critical for ion binding and translocation. The PeNHX3 structure follows the ‘positive-inside’ rule and exhibits a typical physicochemical property of the transporter proteins. Four conserved residues, including Tyr149, Asn187, Asp188, and Arg356, are indentified in the TM4-TM11 assembly region of PeNHX3. Mutagenesis analysis showed that these reserved residues were essential for the function of PeNHX3: Asn187 and Asp188 (forming a ND motif) controlled ion binding and translocation, and Tyr149 and Arg356 compensated helix dipoles in the TM4-TM11 assembly. PeNHX3 mediated Na^+^, K^+^ and Li^+^ transport in a yeast growth assay. Domain-switch analysis shows that TM11 is crucial to Li^+^ transport. The novel features of PeNHX3 in ion binding and translocation are discussed.

## Introduction

Salt stress is one of the most severe environmental factors limiting the productivity of crop plants [1]–[3]. Understanding the mechanisms underlying plant salt tolerance, therefore, is important for developing crop production [4], [5]. Studies have shown that some salt tolerant plants, named halophytes, are capable of surviving in saline soils [6]. Thus, exploring how the halophytes deal with salt is helpful to improve agricultural production [6], [7].

*Populus euphratica* is a well-known tree halophyte that exists in saline desert areas from northwest China to western Morocco [8]. This tree halophyte can tolerate NaCl stress up to 450 mM [9]. Its seedlings accumulated low levels of Na^+^ under salt stress [10]. A recent genomic study found that the genes encoding ion transporters were selectively expanded in the *P. euphratica* genome [11]. These studies suggest that maintaining low levels of salt in the cell is a mechanism for *P. euphratica* to tolerate salt stress. Furthermore, ion transporters controlling cellular salt movement may play critical roles in this tree halophyte under salt stress.

Na^+^,K^+^/H^+^ antiporters (NHX antiporters) are H^+^-coupled cotransporters that transfer the Na^+^ or K^+^ across a membrane in exchange for protons (H^+^) [12], [13]. Their exchange activity is driven by the H^+^ electrochemical gradient generated by the H^+^ pumps such as the plasma membrane H^+^-ATPase or the vacuolar membrane H^+^-ATPase and H^+^-pyrophosphatase [4]. Biochemical and genetic studies have shown that plant NHX antiporters play an important role in salt tolerance [14]–[18]. In Arabidopsis, *Atnhx1* and *Atsos1* mutants are sensitive to salt stress [19], [20]; overexpression of *AtNHX1* and *SOS1/AtNHX7* reduces cytoplasmic Na^+^ content and enhances salt tolerance in Arabidopsis [14], [21], [22]. Further study shows that SOS1 activity is regulated by SOS2 kinase [16], [17], [23], and SOS1 is activated by the removal of a C-terminal auto-inhibitory domain upon phosphorylation by the SOS2/SOS3 complex [24]. AtNHX1 may also be regulated by SOS2 kinase [18]. Also, CaM binds and inhibits the Na^+^/H^+^ antiport activity of AtNHX1 [25]. Recent study shows that AtNHXs may also be involved in pH and K^+^ homeostasis, vesicle trafficking, and plant growth and development [26]–[28]. AtNHX1 and LeNHX2 have a K^+^/H^+^ transport activity and mediate K^+^ compartmentation in vacuoles [20], [29]–[32]. The NHX antiporters in *Ipomoea tricolor* and *Ipomoea Nil* are involved in vacuolar pH regulation; mutation of a NHX gene in *Ipomea Nil* abolishes the colour change in flowers [33], [34]. *nhx1 nhx2* double knockout mutants show significantly reduced growth and abnormal stamens, suggesting their roles in cell expansion and flower development [35]. *nhx1 nhx2* double mutants have reduced vacuolar K^+^ pool, compromised turgor generation for cell expansion, and impaired osmoregulation, suggesting that AtNHX1 and AtNHX2 are critical for cellular K^+^ uptake and stomatal movement [36].

In *P. euphratica*, six NHX genes (*PeNHX1-6*) have been isolated and their ion transport activities have been tested in a yeast growth assay [37]. Using the electrophysiological technique, it has been found that *P. euphratica* has a higher Na^+^/H^+^ antiport activity than its salt-sensitive congener *P. popularis*, suggesting that PeNHXs may function in salt tolerance in the tree halophyte [10]. However, understanding the catalytic mechanisms governing ion transport for the PeNHXs requires a structural model of these antiporters. Up to now, only the crystal structure of EcNhaA from *E. Coli* has been determined within the NHX gene family, and currently there are not any crystal structures available for the eukaryotic NHX antiporters in the database [38], [39].

Homology modeling is a computational approach by which the three-dimensional (3D) structure of a protein (target) is constructed using a protein having identified crystal structure as a template [40], [41]. Since both the bacterial EcNhaA and eukaryotic NHX antiporters have similar function in controlling pH and ion homeostasis, and share a common ancestor and a similar structural fold, hence, it is reasonable to use EcNhaA as a template to predict the structure of the eukaryotic NHX antiporters [42]–. To date, EcNhaA has been used successfully as a template to generate the structures of Human NHXs NHE1 and NHA2 [43], [45]. Landau et al. [43] have constructed a model structure of human NHE1 using EcNhaA as a template, and the predicted structure fitted properly with the results obtained by the previous mutagenesis, inhibitor binding, and NMR studies [43]. Schushan et al. [45] generated a structure of human NHA2 based on EcNhaA, and some key residues involved in ion transport have been identified by a model guided mutagenesis analysis. The experimental and structural analysis revealed a novel structural attributes for NHA2 and suggested a mechanism of antiport different from the previously characterized NhaA- and NHE1-type transporters [45].

In this study, the structural features and catalytic mechanism of PeNHX3 were studied by building a 3D structure. The structure of PeNHX3 was created by homology modeling. Structural characteristics of PeNHX3 were examined by comparing with the crystal structure of EcNhaA. The functions of the conserved residues were analyzed by mutagenesis analysis in yeast. This model can be used to understand the catalytic and regulatory mechanisms of PeNHX3 in the tree halophyte *P. euphratica* under salt stress.

## Materials and Methods

### Fold identification, TM helix prediction, and pairwise alignment

The Pfam database was used to analyze protein families [46]. The fold- recognition server FFAS03 was used to identify protein folds [47]. Using the sequence of PeNHX3, FFAS03 identified the structure of EcNhaA (PDB ID 1ZCD) with a significant score of −121. Based on the FFAS03 analysis, scores lower than −9.5 indicate significant similarity between the proteins; thus, EcNhaA is the best template for PeNHX3.

HMMTOP [48] and TMHMM [49] were used to predict TM helices. The pairwise alignments were computed with the FFAS03 server [47] and the FUGUE server [50].

### 3D model construction by homology modeling

The homology modeling program SwissPdb viewer was used to build the 3D model of PeNHX3 [51]. The template EcNhaA (PDB ID 1ZCD) and the pairwise alignment between PeNHX3 and EcNhaA (Figure S1) were used to generate the structural model of the TM domains (residues 17–799).

### Yeast Strains, media, and growth conditions

*Saccharomyces cerevisiae* strain AXT3 (*ena1-4Δ::HIS3, nha1Δ::LEU2, nhx1Δ:: TRP1*) was a gift from Dr. Jose M. Pardo [52]–[54]. This strain was the derivative of W303-1B. Untransformed strains were grown at 30°C in YPD medium (1% yeast extract, 2% peptone and 2% glucose). Transformation of yeast cells was performed by the lithium acetate method [55]. After transformation, strains were grown on selective Hartwell's complete (SC) medium or APG medium (10mM arginine, 8mM phosphoric acid, 2 mM MgSO_4_, 1 mM KCl, 0.2 mM CaCl_2_, 2% glucose, and trace minerals and vitamins). NaCl, LiCl, KCl, or hygromycin B was added to the medium. Drop test media contained 20 mM MES, and pH was adjusted with arginine [56] or to acidic pH values with phosphoric acid [57].

### Generation of the point mutants of PeNHX3

pDR196-PeNHX3 was used as a template to generate the point mutants. The site-directed mutagenesis was performed by Quikchange mutagenesis. The mutations were GAT to GCT, GAA, or AAT (D188A, D188E, or D188N); AAT to GCT, GAT, GAA, or CAA (N187A, N187D, N187E, or N187Q); TAT to GCT, GAT, AAT, GAA, or CAA (Y149A, Y149D, Y149N, Y149E, or Y149Q); and AGA to GCT, AAA, or CAA (R356A, R356K, or R356Q), respectively. For the yeast expression assay, these point mutants of PeNHX3 were recombined into the yeast vector pDR196.

### Functional expression of PeNHX3 in yeast

The *AtNHX2* gene, ordered from ABRC, was cloned into the yeast expression vector pDR196 with the promoter PMA1. The empty vector pDR196 was received from Dr. John Ward as a gift. To clone *PeNHX3*, gene fragments were amplified by PCR from *Populus euphratica* cDNA using the following primers: PeNHX3 (5′- CCGGAATTCATGGAAGCTTATATGAGTTC -3′ and 5′- CCGCTCGAGTCAATGCCATTGATTCTGAG -3′). To clone *ScNHX1*, gene fragments were amplified by PCR from the genomic DNA isolated from the *Saccharomyces cerevisiae* strain BJ3505 using the following primers: ScNHX1 (5′-CGCGTCGACATGCTATCCAAGGTATTGC-3′ and 5′-CCGCTCGAGCTAGTGGTTTTGGGAAGAG-3′). The PCR fragments were cloned into the plasmid pDR196. All gene fragments were verified by sequencing.

All plasmids were transformed into the yeast strain AXT3, and the empty vector pDR196 was transformed into the same yeast strains as a control. For the stress tolerance tests, yeast cells were normalized in water to A_600_ of 0.12. 4 µl aliquots of each 10-fold serial dilution were spotted onto AP plates supplemented with KCl, or YPD plates supplemented with NaCl or LiCl as indicated, and incubated at 30°C for 3 days. Resistance to hygromycin B was assayed in YPD medium.

The salt-sensitive growth of the yeast cells expressing PeNHX3 and its mutants was measured in liquid APG media. We prepared a preculture by growing the previously propagated yeast in 2 ml APG media overnight at 30°C. The cells were centrifuged, washed once with water, and resuspended in water (OD_600_≈0.65). Inoculate 0.2 ml APG medium in a 96-well microplate with 4 µl of the seed culture. After incubation at 30°C for 48 h, cultures were gently resuspended, and the OD_600_ was recorded on a Thermo Scientific Varioskan Flash plate reader.

### Domain-switch analysis

The AtNHX1 gene, ordered from ABRC, was cloned into the yeast expression vector pDR196 with the promoter PMA1. A fragment of AtNHX1 CDS beginning from the start codon ATG to T1092 was subcloned into the vector pDR196. Two fragments of PeNHX3 CDS, C537 (A1102-A1638) and C399 (A1240-A1638), which encode two peptides of the C-terminal domain of PeNHX3, were fused with the AtNHX1 fragment to produce the fusion proteins of AtNHX1-PeNHX3-C537 and AtNHX1-PeNHX3-C399, respectively.

### Sequence alignment analysis

The predicted amino acid sequences of PeNHX3 and AtNHX1 were collected from the GenBank (http://www.ncbi.nlm.nih.gov). Proteins were compared by multiple alignments using the ClustalX 2.1 method (Larkin et al., 2007). The alignment is based on the complete amino acid sequences.

## Results

### TM helix assignment of PeNHX3

As the only NHX antiporter having a crystal structure, EcNhaA has been used as a template to build the homology modelings for Human NHXs NHE1 and NHA2 [43], [45]. In this study, we are interested in generating a model structure for the *P. euphratica* NHX antiporter 3 (PeNHX3) using EcNhaA as a template. We found that both PeNHX3 and EcNhaA belonged to the same phylogenetic clan as detected by the Pfam database [46]. Using the PeNHX3 sequence as a target, we identified EcNhaA as the closest homologue by the fold-recognition server FFAS03 [47].These results suggest that both PeNHX3 and EcNhaA are evolutionarily related and may share a similar fold.

**Figure 1:**
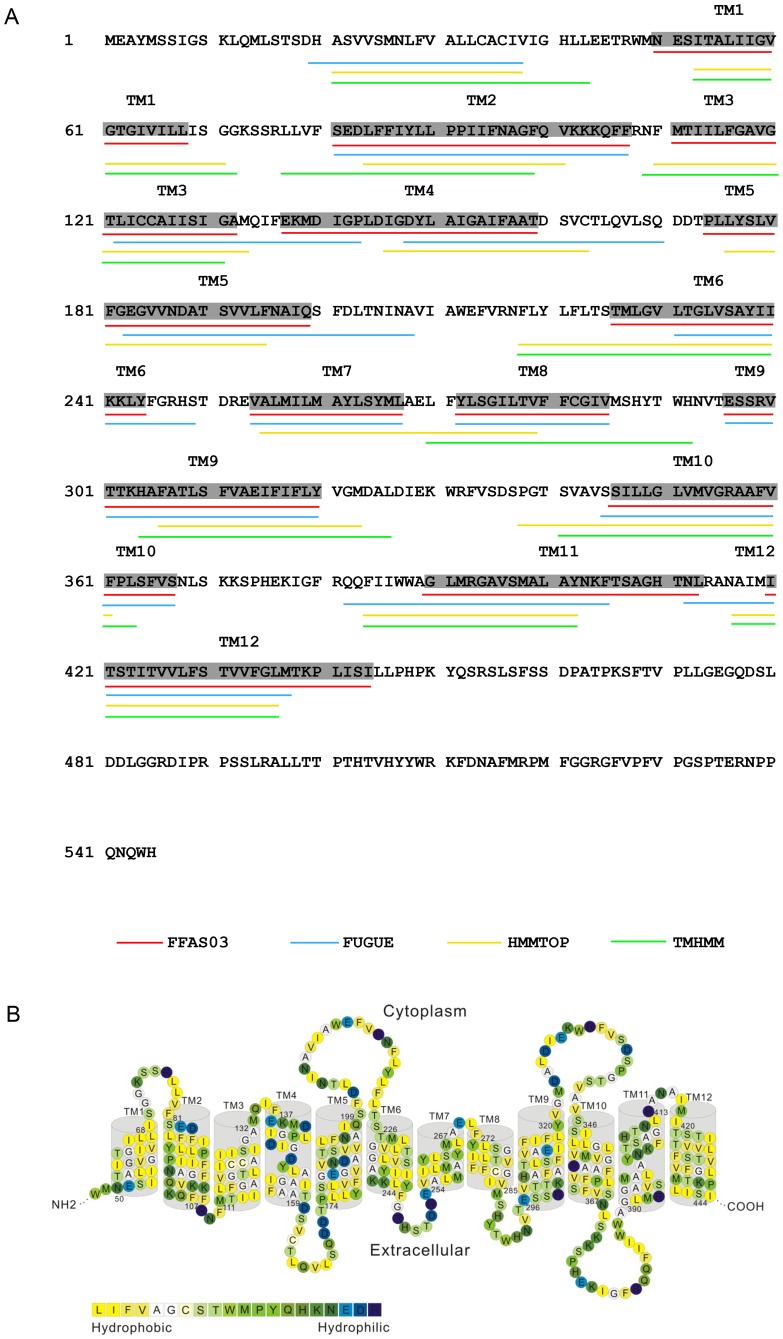
The TM segments and topology of PeNHX3. A. The TM helices were analyzed with FFAS03, FUGUE, HMMTOP, and TMHMM. The TM helix numbers are marked at the corresponding regions. The TM boundaries for structure modeling are highlighted in gray. B. The TM topology of PeNHX3. Amino acid residues are colored based on the hydrophobicity scale of Kessel and Ben-Tal (Schushan et al., 2010).

Since we cannot use the standard methods to align the sequences between EcNhaA and PeNHX3 due to the low sequence identity between these two proteins (below 10%) (data not shown), we used a combined approach to obtain the alignment between the target and template sequences. The pairwise alignments were constructed by the FFAS03 [47] and FUGUE algorithms [50] (Figure 1A). In addition, the TM and secondary-structure prediction algorithms and hydrophobicity analysis were included to assist the selection of the TM helix boundaries (Figure 1A).

FFAS (the fold and function assignment system) is a profile-profile comparison algorithm that has a high sensitivity and alignment accuracy compared with other methods [47]. Therefore, FFAS is frequently used for the detection of remote homologies. In our analysis, we found that the FFAS03 alignments match the results of FUGUE, HMMTOP, and TMHMM (Figure 1A). Thus, we utilized the helix assignment produced by the FFAS03 to establish the boundaries of the TM helices for PeNHX3 in this study (Figure 1A, 1B).

Gaps were found in TM segments 2–6 of PeNHX3 in the FFAS03 alignment. We manually removed these gaps and assigned the helices according to the TM segments of EcNhaA (Figure 1A).

### 3D model building of PeNHX3

To build the 3D structure of PeNGX3, we aligned the TM segments of PeNHX3 with EcNhaA based on the FFAS03 prediction. We then used this alignment and the EcNhaA template to construct a 3D model of PeNHX3 with SwissPdb viewer [51].

**Figure 2:**
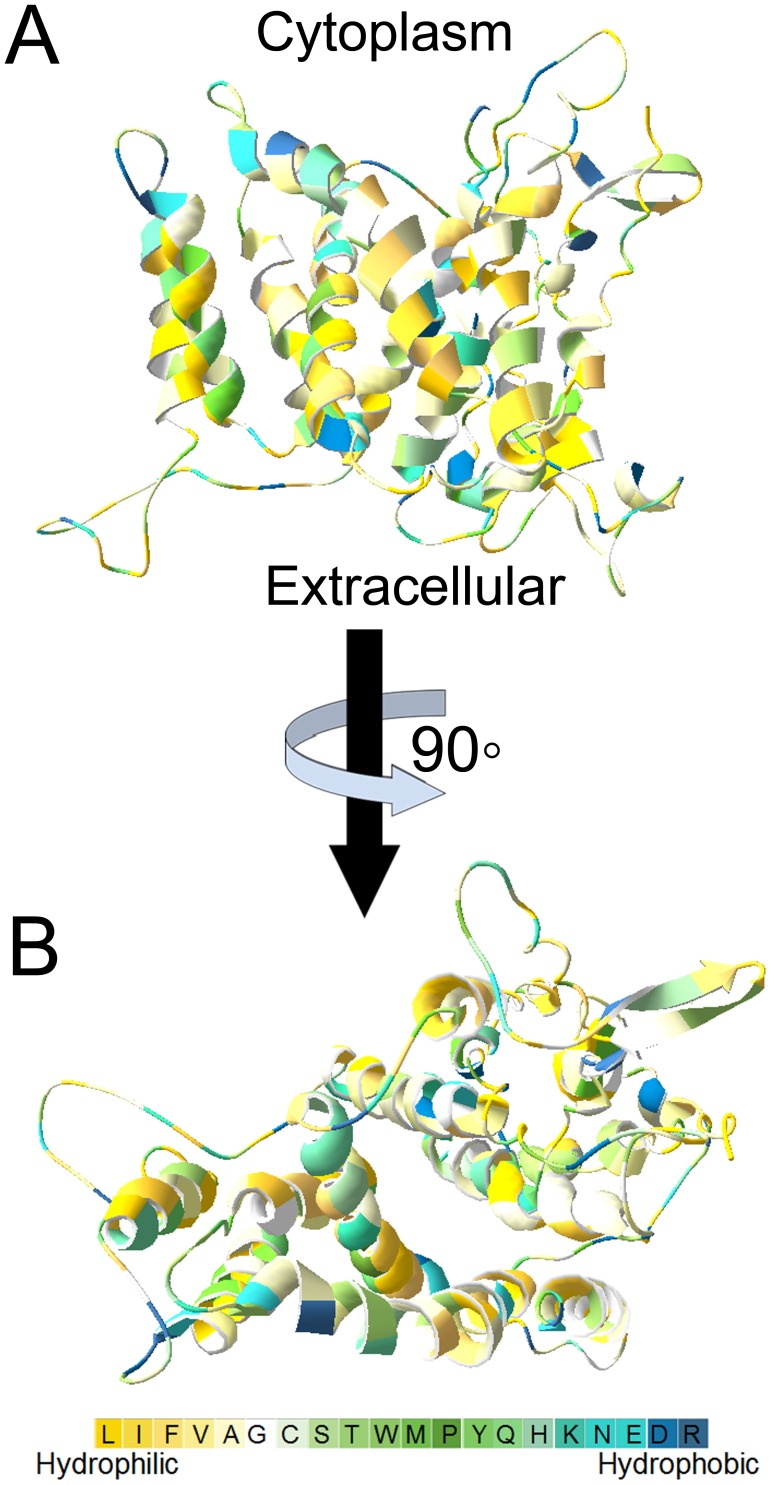
Hydrophilic residues are localized in the core of PeNHX3. The PeNHX3 model is colored based on the hydrophobicity scale of Kessel and Ben-Tal (Schushan et al., 2010). A. A side view parallel to the membrane with the intracellular side facing upward. B. A top view from the extracellular side.

As shown in Figure 2A, the PeNHX3 model exhibited the typical TM4-TM11 assembly that has been identified in the structures of the NHX antiporters (EcNhaA, NHE1 and NHA2) [38], [39], [43], [45]. In the PeNHX3 model, the TM4 and TM11 segments unwound to form extended peptides in the center of the helix and crossed each other in the middle of the membrane. The TM4-M11 assembly was embedded between TM1, TM3, TM5, TM10, and TM12 (Figure 2A).

In the PeNHX3 model, the polar residues were clustered either in the core structures or on extramembrane loops while the hydrophobic residues were facing the membrane (Figure 2A), displaying a typical physicochemical property of the transporter proteins [45].

**Figure 3:**
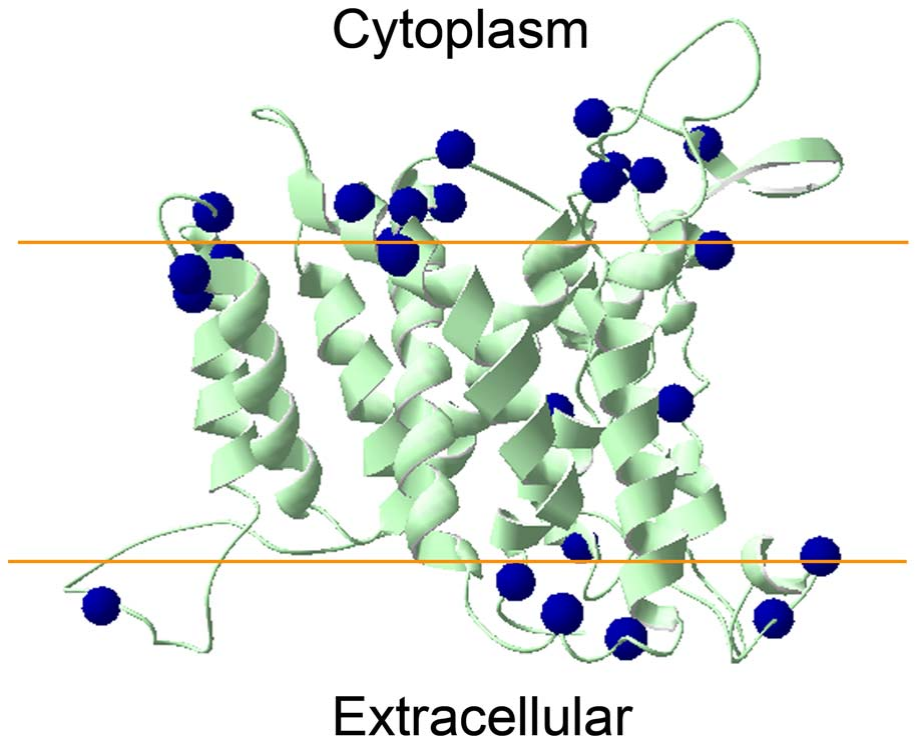
The PeNHX3 model follows the ‘positive-inside’ rule. The cytoplasmic side is at the top. The approximate boundaries of the hydrocarbon region of the membrane are shown in orange. The Cα atoms of lysines and arginines in the PeNHX3 model are shown as blue spheres.

The ‘positive-inside’ rule is an empirical observation that has been used to evaluate the structural model of membrane proteins [45]. This rule suggests that the intracellular regions are enriched with positively charged residues (lysine and arginine) compared with the extracellular regions in the structure of membrane proteins [45], [58], [59]. This ‘positive-inside’ rule has been well demonstrated by the structures of the NHX antiporters EcNhaA, NHE1 and NHA2 [38], [43], [45]. We are interested in understanding if our PeNHX3 model fits the ‘positive-inside’ rule. As shown in Figure 3, of the 25 lysines and arginines in the PeNHX3 model, 18 positively charged residues were localized in the cytoplasmic side whereas 7 in the extracellular side of the membrane (Figure 3), suggesting that our PeNHX3 model follows the ‘positive-inside’ rule.

### PeNHX3 mediates Na+, K+ and Li+ transport in yeast

**Figure 4:**
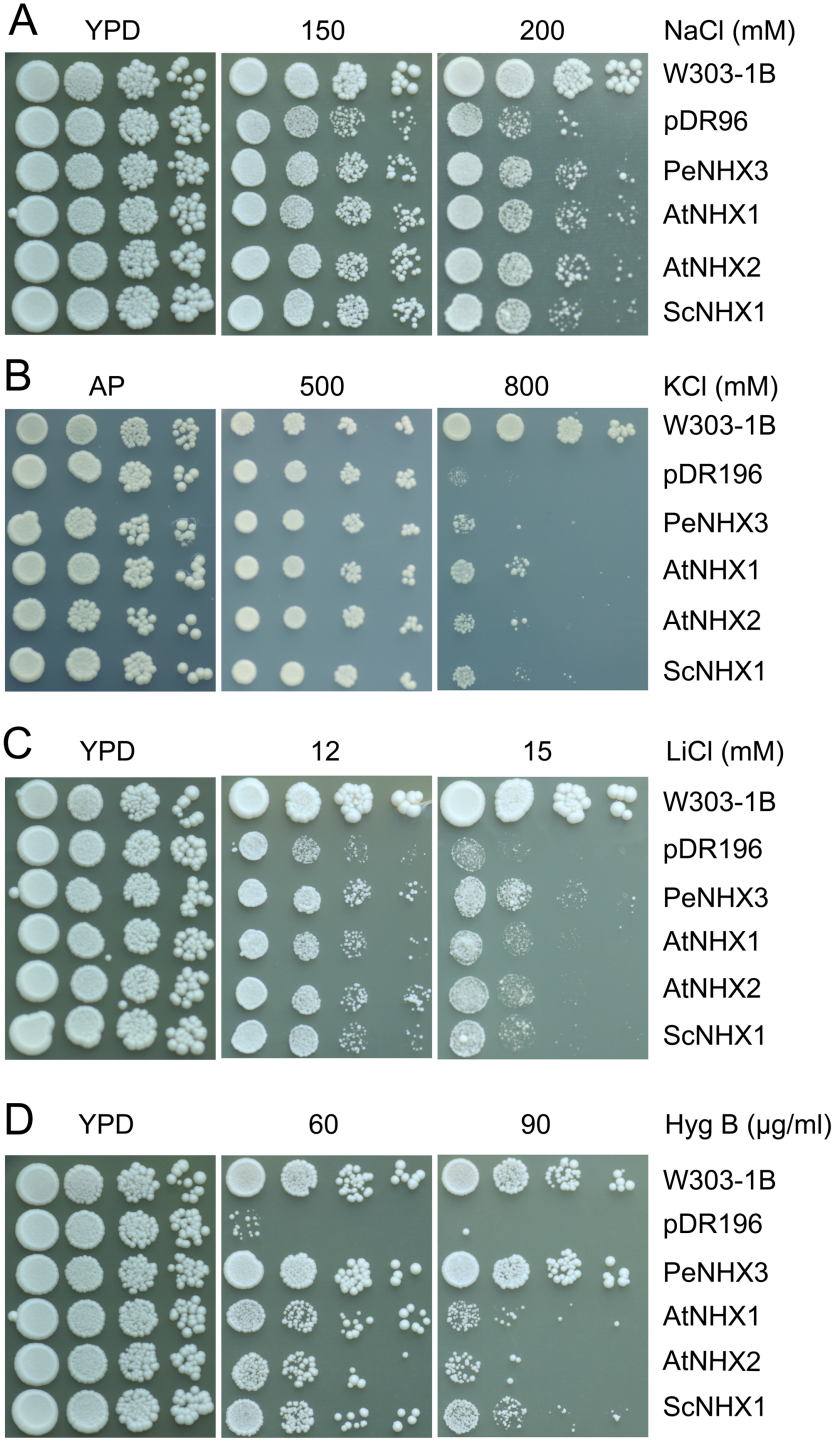
PeNHX3 facilitate Na^+^, K^+^ and Li^+^ transport and confer tolerance to hygromycin B in yeast. The cDNAs of PeNHX3, AtNHX2, AtNHX2, and ScNHX1 were subcloned into the yeast expression vector pDR196 and transformed into the AXT3 mutant (*ena1-4 nha1 nhx1*). The yeast strains were grown overnight in AP or YPD medium. Cells were normalized in water to A600 of 0.12. Aliquos (4 µL) from the normalized yeast cultures or 10-fold serial dilutions were spotted onto AP plates supplemented with KCl (B) or onto YPD plates with NaCl (A), LiCl (C), or Hygromycin B (D). The strains were grown at 30°C for 3 days.

PeNHX3 has been reported to be tolerant to salt, high K^+^ or Li^+^ stress in a yeast growth assay [37]. However, PeNHX3 transport activity was not compared with the plant or yeast NHX antiporters. Te better understand the function of PeNHX3, we compared its activity with AtNHX1, AtNHX2 and ScNHX1 using a yeast expression system. *PeNHX3* gene was cloned into the yeast vector pDR196 and expressed in a *Saccharomyces cerevisiae* strain AXT3. Strain AXT3 is sensitive to salt and to high K^+^ since it lacks the functional plasma membrane Na^+^-ATPases (ScENA1-4), plasma membrane Na^+^,K^+^/H^+^ antiporter ScNHA1, and vacuolar Na^+^,K^+^/H^+^ antiporter ScNHX1. The transformed yeast was grown on Arg phosphate (AP) or YPD medium with high levels of KCl, NaCl or LiCl (Figure 4). *AXT3* mutants did not grow well in the presence of 200 mM NaCl while the *nhx1*-positive strains (W303-1B) grew dynamically (Figure 4A). The AXT3 strain expressing *PeNHX3* recovered tolerance to salt stress. In addition, expression of PeNHX3 conferred resistance to high K^+^, but to a lesser extent compared to the strains expressing AtNHX1, AtNHX2, or ScNHX1 (Figure 4B). Interestingly, PeNHX3 was highly tolerant to Li^+^ stress (Figure 4C). While *AXT3* mutants failed to grow in the medium containing hygromicin B (90 µg/ml), PeNHX3 greatly enhanced tolerance to the drug hygromicin B. PeNHX3 tolerance to hygromicin B was much stronger than AtNHX1, AtNHX2, or ScNHX1 (Figure 4D), suggesting that PeNHX3 may have a significant impact on endosomal compartments. These results suggest that PeNHX3 is similar to both the Arabidopsis and yeast NHX antiporters in that all NHX antiporters facilitate Na^+^ and K^+^ homeostasis. However, unlike its Arabidopsis and yeast counterparts, PeNHX3 may play a vigorous role in Li^+^ detoxicity and endosomal trafficking.

### TM11 may control the Li^+^ transport activity of PeNHX3

**Figure 5:**
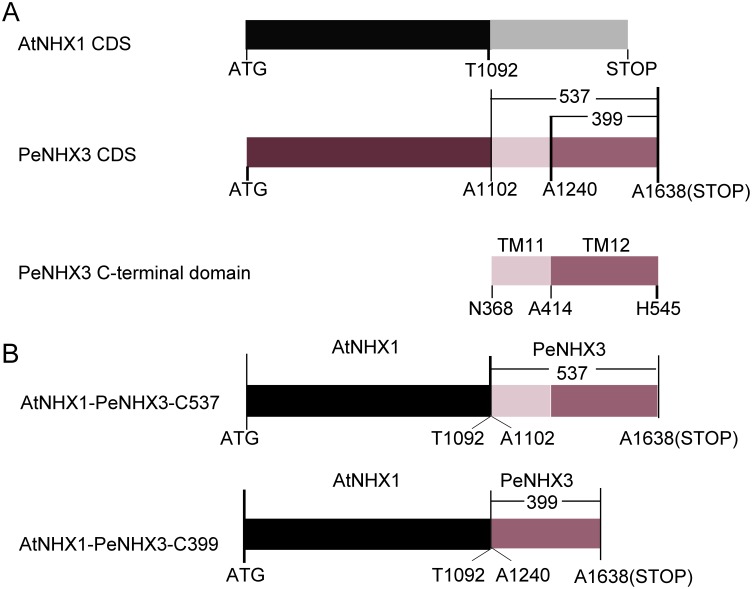
Schematic diagram of the yeast expression constructs for the domain-switch analysis. (A) The CDS of AtNHX1and PeNHX3, and the C-terminal domain of PeNHX3. (B) The constructs of AtNHX1-PeNHX3-C537 and AtNHX1-PeNHX3-C399. Construct diagrams are not drawn to scale.

**Figure 6:**
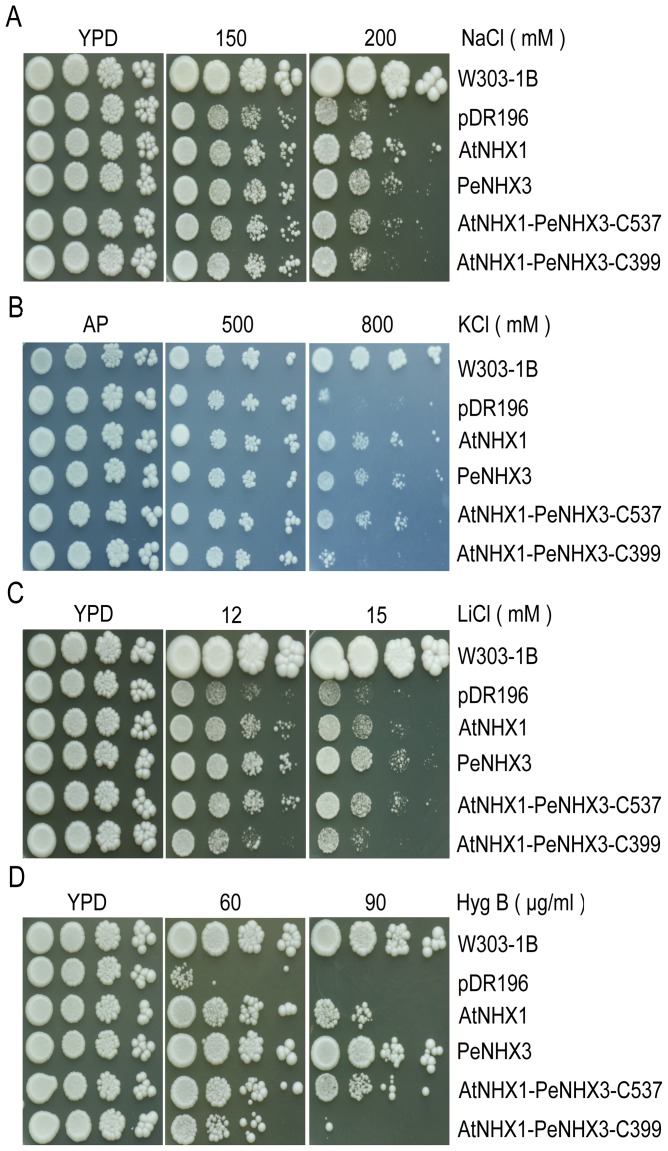
TM11 is crucial to Li^+^ transport in PeNHX3. The yeast strains were grown overnight in AP medium. Cells were normalized in water to A600 of 0.12 Aliquos (4 µL) and 10-fold serial dilutions were spotted on AP plates supplemented with KCl (B) or YPD plates with NaCl (A), LiCl (C), Hyg B (D) as indicated on each panel. The strains were grown at 30°C for 3 days.

To investigate the mechanism controlling Li^+^ transport by PeNHX3, we performed the domain-switch assays. We first compared the sequence of PeNHX3 with that of AtNHX1 by alignment. The alignment shows that the region ranging from Asn368 to the C-terminal end of PeNHX3 is less conserved compared with the remaining parts of the protein sequence (Figure S2). Thus, the less conserved sequence in this C-terminal domain suggests that the C-terminal region may be responsible for the diversified Li^+^ transport activity between PeNHX3 and AtNHX1. Further analysis shows that this diversified C-terminal region of PeNHX3 mainly contains two transmembrane domains, TM11 and TM12 (Figure 1A, 1B). Since ion transport is primarily catalyzed by the transmembrane domains of the antiporters, we reason that these two transmembrane domains (TM11 and TM12) may be the critical players of the higher Li^+^ transport in PeNHX3. To test this hypothesis, we conducted the domain-switch analysis by exchanging these two transmembrane domains of PeNHX3 with the corresponding regions of AtNHX1. We generated two constructs that produce the fusion proteins of AtNHX1 and PeNHX3: AtNHX1-PeNHX3-C537 and AtNHX1-PeNHX3-C399, respectively (Figure 5A, 5B). AtNHX1-PeNHX3-C537 contains a peptide ranging from Asn368 to His545 (the end of PeNHX3 amino acid sequence), including both the TM11 and TM12 of PeNHX3 (Figure 5B). On the other hand, AtNHX1-PeNHX3-C399 contains a shorter peptide ranging from Ala414 to His545, including only TM12 of PeNHX3 (Figure 5B). The ion transport activity of these two AtNHX1-PeNHX3 fusion proteins was tested in yeast (Figure 6). Interestingly, AtNHX1-PeNHX3-C537 displayed a higher capacity in conferring Li^+^ tolerance than its parent protein AtNHX1, and reached the same level as PeNHX3 (Figure 6C). In contrast, AtNHX1-PeNHX3-C399 had a much lower ability in recovering Li^+^ tolerance compared with the parent protein AtNHX1(Figure 6C). These results suggest that TM11 may play a central role in catalyzing Li^+^ transport in PeNHX3. In addition, AtNHX1-PeNHX3-C537 conferred tolerance to salt tress, high K^+^ and hygromicin B, while AtNHX1-PeNHX3-C399 did not (Figure 6A, 6B, 6D), suggesting that TM11 is also crucial to the transport of Na^+^, K^+^ and hygromicin B in PeNHX3.

### ND motif is essential for the transport activity

**Figure 7:**
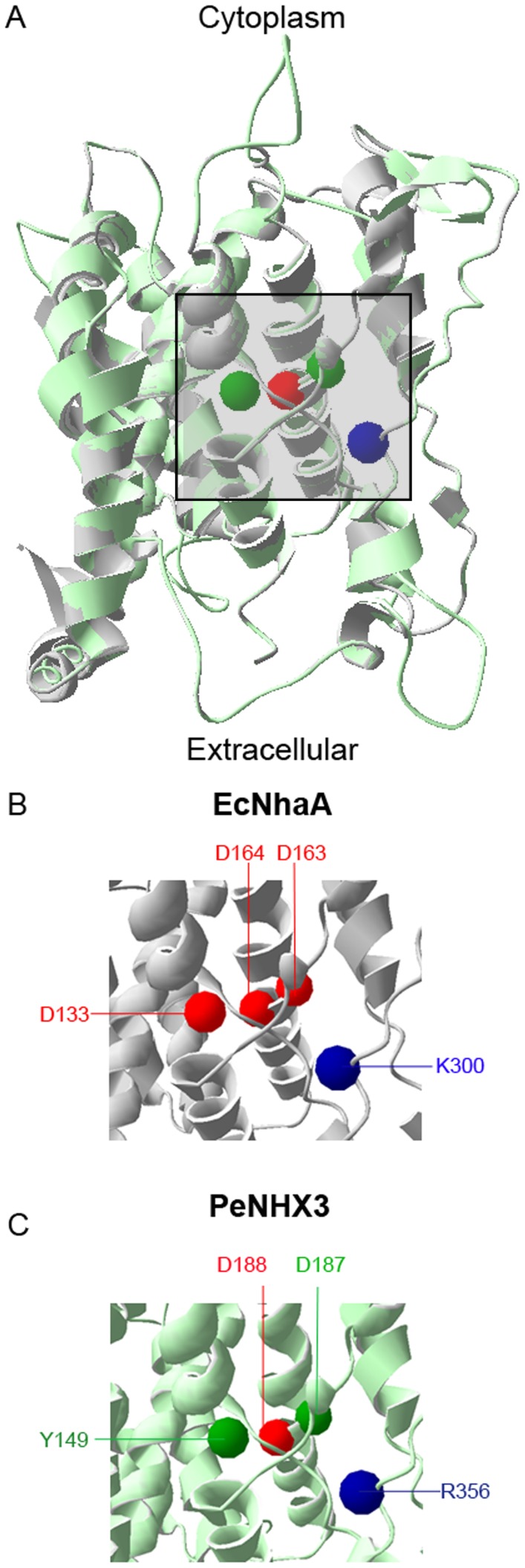
Comparison of the four conserved residues in the TM4-TM11 assembly regions of PeNHX3 and EcNhaA. The cytoplasmic side is at the top and TM12 was omitted for clarity in all panels. The Cα atoms of the four conserved titratable residues in the TM4-TM11 assembly region are shown as spheres. A. The predicated structure of PeNHX3 (light green) is aligned to the crystal structure of EcNhaA (gray) (Hunte et al., 2005). The center of the TM4-TM11 assembly and flanking region is marked by a square. B and C. The marked regions of EcNhaA and PeNHX3.

**Figure 8:**
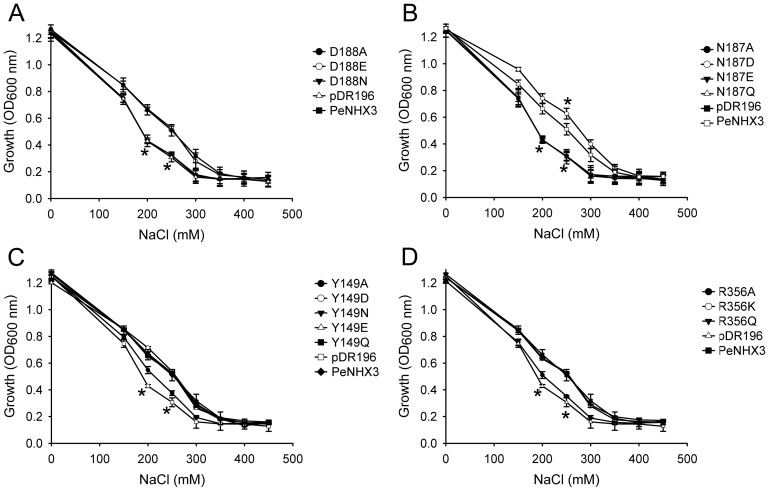
Functional analysis of the conserved residues in the TM4-TM11 assembly region of PeNHX3 (dosage response). The cDNAs of the wild type PeNHX3 and the PeNHX3 mutants were subcloned into the yeast expression vector pDR196 and transformed into the AXT3 mutant (*ena1-4 nha1 nhx1*). Yeast cells were grown in APG media containing different concentrations of NaCl. Yeast growth was determined at 600 nm (OD_600_) after 48 h at 30°C. Data are the average of three experiments. Asterisks indicate significant difference (P≤0.05; *t* test).

**Figure 9:**
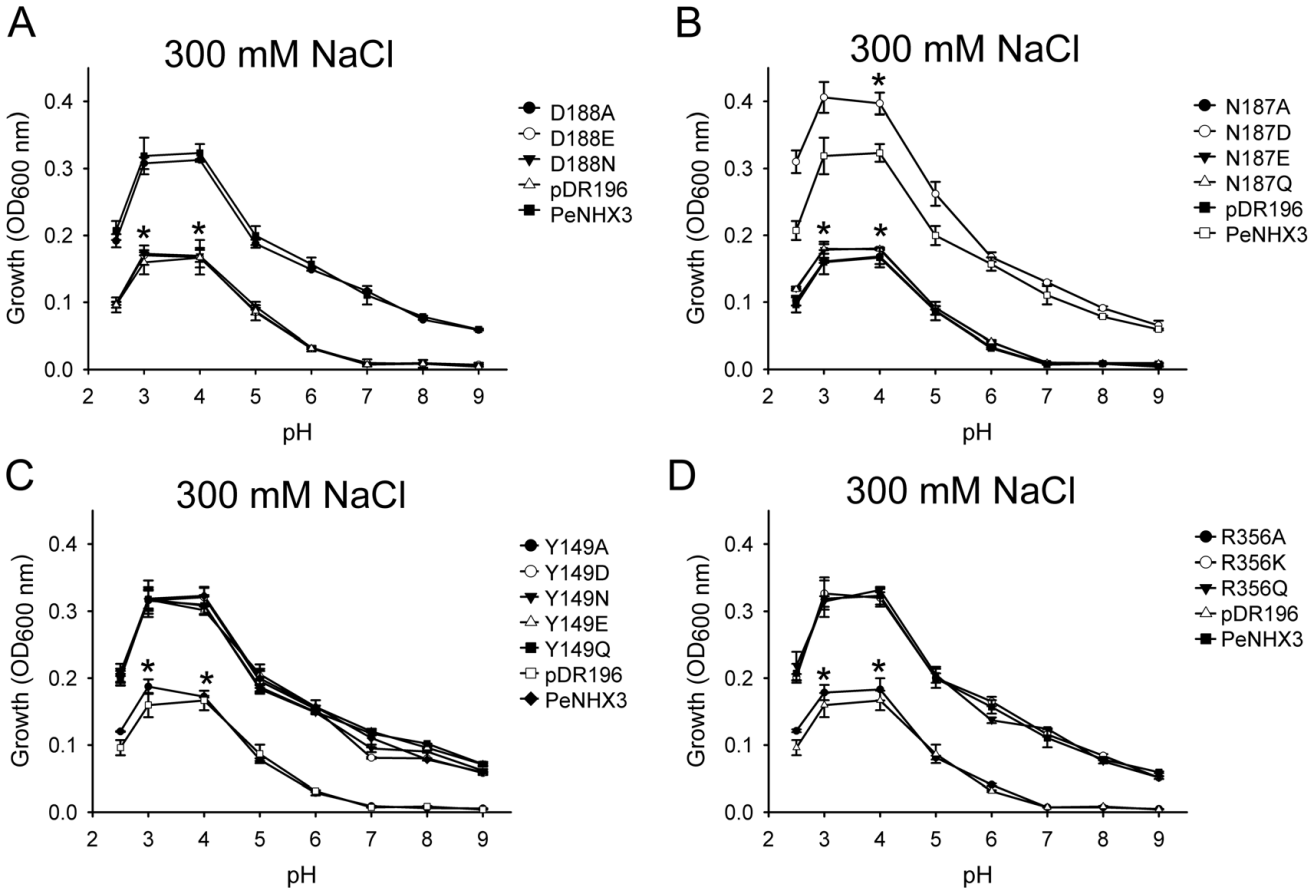
Functional analysis of the conserved residues in the TM4-TM11 assembly region of PeNHX3 (pH profile). NaCl concentration was kept at 300Figure 6. Asterisks indicate significant difference (P≤0.05; *t* test).

Structural studies have shown that the CPA2 antiporter EcNhaA has two sequential Asp residues, Asp163 and Asp164, in TM5 (Figure 7A, 7B) [38], [39]. Asp163 and Asp164, which form a DD motif, localize in the TM4-TM11 assembly region of EcNhaA. The DD motif has been demonstrated to be involved in ion binding and translocation, and thus is essential for the transport activity [38], [39], [43], [45]. In CPA1 family, however, the corresponding residues are Asn and Asp that form a ND motif. Study has shown that the ND motif corresponding to Asn266 and Asp267 in NHE1, a CPA1 antiporter, has a similar function as the DD motif in ion binding and translocation [45]. Similar to its CPA1 counterpart NHE1, we identified a ND motif composed of the residues Asn187 and Asp188 in the TM5 of PeNHX3, which are homologous to Asp163 and Asp164 of EcNhaA, respectively (Figure 7C). To investigate the function of the ND motif in PeNHX3, we conducted site-directed mutagenesis by individually replacing Asn187 with glutamine, glutamate, or alanine, and replacing Asp188 with glutamate, asparagine, or alanine, respectively. The transport activities of the mutant genes were determined in a yeast expression system (Figure 8, 9). The transformed yeast was grown in medium with different NaCl concentrations or pH values (Figure 8, 9). The results showed that except for D188E, all the point mutants did not rescue the growth of the salt sensitive strain under salt stress, suggesting a specific requirement for an asparagine residue at Asn187 and an acidic residue at Asp188 locations, respectively.

Studies have shown that the mutant proteins lost their transport activities and failed the functional complementation test when the DD motif in CPA2 antipoters NHA2 or EcNhaA was substituted to an ND motif [45], [60]. We are interested in exploring if the DD motif is functioning in the CPA1 antiporters by generating the N187D mutant in PeNHX3. Interestingly, the N187D mutant, in which the original ND motif was transformed into an DD motif, grew even faster than the wild type yeast under salt stress (Figure 8, 9), suggesting that the DD motif functions equally well as the ND motif in PeNHX3.

### Tyr 149 and Arg356 are vital for charge compensation

One of the unique features of the NhaA structure is that its TM4 and TM11 helices are discontinuous and interrupted by an extended chain [38], [39]. These extended chains in TM4 and TM11 helices are flanked by partial charges formed at the N- and C-termini of the respective small helices connecting them. These partial charges are compensated by the negatively charged residue Asp133 of TM4 and the positively charged residue Lys300 of TM10, respectively (Figure 7A, 7B) [38], [39]. In our PeNHX3 model, we identified Arg356 in TM10 as the equivalent of Lys300 in EcNhaA (Figure 7C). However, we did not find a negatively charged residue in the position corresponding to Asp133 of EcNhaA, instead we found a polar uncharged residue Tyr149 at this site in PeNHX3 (Figure 7C). In order to understand if these two residues are functioning in compensating the helix dipoles in the TM4-TM11 assembly, we performed mutagenesis analysis (Figure 8, 9). Interestingly, R356K fully recovered yeast growth under salt stress while R356A failed to rescue salt-tolerant growth (Figure 8, 9), suggesting that positive charges are necessary for the charge compensation at the site of Arg356. Moreover, R356Q rescued yeast growth to the same level as the wild type strain (Figure 8, 9), suggesting that the partial positive charge of the polar residue glutamine can stabilize the partial negative dipoles of TM4 and TM11 in PeNHX3.

In case of Tyr149, while Y149A did not recover yeast growth, all other mutants, including Y149D, Y149N, Y149E, and Y149Q, fully rescued yeast growth under salt stress (Figure 8, 9), suggesting that either the negative charges of Asparate and glutamate or the partial negative charges of asparagine and glutamine can stabilize the partial positive dipoles of TM4 and TM11 in PeNHX3.

## Discussion

### PeNHX3 may function in salt stress and Li+ detoxification in *P. euphratica*

A study has shown that PeNHX3 is a tonoplast antiporter that mediates Na^+^, K^+^, and Li^+^ transport [37]. PeNHX3 gene expression was highly induced in the roots, stems, and leaves of *P. euphratica* under salt stress [37]. Our results showed that PeNHX3 recovered yeast growth under salt stress (Figure 4A). We also showed that PeNHX3 had a higher transport activity for Li^+^ relative to the Arabidopsis and yeast antiporters (Figure 4C). Thus, these results suggest that PeNHX3 may play an important role in salt stress and Li^+^ detoxification in *P. euphratica*. Nevertheless, PeNHX3 showed a lower K^+^ transport activity compared to Na^+^ transport (Figure 4A, 4B), suggesting that PeNHX3 may have a higher affinity for Na^+^ over K^+^.

Moreover, we showed that PeNHX3 conferred resistance to hygromycin B in yeast growth (Figure 4D). The change in sensitivity to the drug hygromycin B represents the alteration in membrane potential and membrane trafficking. It is increasingly evident that Na^+^,K^+^/H^+^ antiporters play an important role in membrane trafficking [28]. Studies have shown that yeast ScNhx1p is vital for vacuole fusion [61], [62] and vesicle trafficking out of the late endosome [63]–[65]. Human NHE8 is critical for maintaining endosomal structure and for the regulation of protein sorting [66]. In Arabidopsis, AtCHX17 and AtCHX21 mediate protein sorting [56]. AtNHX5 and AtNHX6 are involved in vacuolar trafficking [67]. Therefore, PeNHX3 may also play an important role in membrane trafficking in *P. euphratica*.

The high Li^+^ transport activity for PeNHX3 revealed in our study is unanticipated. We further analyzed the catalyzing mechanism underlying Li^+^ transport in PeNHX3 using the domain-switch technique. We obtained two fusion proteins of AtNHX1 and PeNHX3 by exchanging two peptides from the C-terminal domain of PeNHX3 with the corresponding regions of AtNHX1 (Figure 5A, 5B). The results showed that the fusion protein AtNHX1-PeNHX3-C537, which contains the TM11 and TM12 of PeNHX3, significantly improved yeast growth under Li^+^ stress, whereas the fusion protein AtNHX1-PeNHX3-C399 that contains only TM12 did not show any capability in conferring Li^+^ tolerance in yeast (Figure 6C). Moreover, we found that AtNHX1-PeNHX3-C537 also improved tolerance to salt stress, high K^+^ and hygromicin B, but AtNHX1-PeNHX3-C399 did not (Figure 6A, 6B, 6D). Taken together, these results suggest that the TM11 may play an important role in catalyzing Li^+^ and other ion transport in PeNHX3. However, our results are preliminary, and more studies, including site mutational analysis, are needed to understand the mechanism controlling Li^+^ transport in PeNHX3 in yeast. In addition, it will be interesting to investigate the *in vivo* function of PeNHX3 in Li^+^ transport and detoxification by complementing Arabidopsis mutants under stress conditions.

### The TM4-TM11 assembly is critical for maintaining the structure and ion transport in PeNHX3

The unique feature of the crystal structure of EcNhaA is that there is an assembly of the TM4 and TM11 segments lying in its structural core, where the two helices cross each other in the middle of the membrane [38]. In the TM4-TM11 assembly, the helices are unwinding to form extended peptides in the center of the helix, providing structural basis for the ion-binding site [38]. The assembly forms dipoles that are charge-compensated and stabilized by Asp133 in TM4 and Lys300 in TM10, respectively [38] (Figure 7A, 7B). In addition, the DD motif containing Asp163 and Asp164 in TM5, which localizes in the TM4-TM11 assembly region of EcNhaA, is involved in ion binding, translocation, and transport activity (Figure 7A, 7B) [38], [39]. Interestingly, both the model structures of NHE1 and NHA2 contain a similar TM4-TM11 assembly as well as the conserved residues in their structural cores [43], [45]. Mutagenesis analysis demonstrated that the conserved residues in both NHE1 and NHA2 were vital for ion binding and partial charge compensation [43], [45]. In our PeNHX3 model, we also indentified a TM4-TM11 assembly that contained extended peptides in their helices (Figure 7C). Four conserved residues, Asn187 (in TM5), Asp 188 (in TM5), Tyr 149 (in TM4) and Arg356 (in TM10), which are the equivalents of Asp163, Asp164, Asp133, and Lys300 in EcNhaA, respectively, have been identified in the PeNHX3 model (Figure 7). Mutagenesis analysis showed that these four conserved residues were essential for ion transport in PeNHX3 (Figure 8, 9). Thus, these results suggest that the TM4-TM11 assembly, a structural core common to the NHX antiporters, may play important roles in ion binding and transport in PeNHX3.

An alternating access mechanism of ion transport has been suggested for EcNhaA [38], [39]. In this hypothesis, EcNhaA forms two funnels that lay the path for ion-transport. One funnel opens to the cytoplasm while the other to the periplasm. However, these two funnels do not form a continuous pore, but are separated by densely packed non-polar residues in the middle of the membrane. Thus, conformational alterations are required to switch the ion-binding site between inward and outward orientations. The TM4-TM11 assembly may provide the structural basis for the alternating access mechanism of ion transport, in which the alternating conformation of the ion binding site is induced at the bottom of the funnels upon pH activation [38], [39]. Since PeNHX3 displays a similar TM4-TM11 assembly in its model structure, we thus suggest that PeNHX3 may catalyze its ion transport with the alternating access mechanism.

### Asn187 and Asp 188 forms a ND motif that is crucial for ion binding and ion translocation in PeNHX

Studies have shown that the DD motif of CPA2 NHXs or the ND motif of CPA1 NHXs plays an important role in ion transport [43], [45]. The DD or ND motif, which is localized close to the TM4-TM11 assembly of the NHX structures, is involved in cation binding and thus is essential to the ion transport of NHX antiporters [43], [45]. Our mutagenesis studies showed that the ND motif of PeNHX3, which is composed of the residues Asn187 and Asp188 in the TM5, was essential for yeast growth under salt stress (Figure 8, 9), suggesting that the ND motif of PeNHX3 plays an important role in ion transport.

An interesting question to ask: is the DD or ND motif exchangeable in CPA1 and CPA2 antiporters? Studies have shown that in NHA2, a CPA2 NHX, the ion transport activity was completely inhibited when its DD motif was mutated into ND motif [45], suggesting that the DD motif is strictly required for CPA2 NHXs. However, our experiments showed that yeast growth was not affected when the ND motif of PeNHX3 was replaced by a DD motif (Figure 8, 9), suggesting that the DD motif is fully functional in CPA1 NHXs.

Since both the DD and ND motifs work equally well in CPA1 NHXs, then why CPA1 NHXs retain a ND motif but not a DD motif? Or in other words, what is the function of the ND motif in CPA1 NHXs? Landau et al. [43] proposed that the ND motif in CPA1 controls the Na^+^:H^+^ stoichiometry of their transport activities. They suggested that Asp164 in EcNhaA or Asp267 in NHE1 serves to alternately bind Na^+^ or H^+^. While Asp163 in EcNhaA binds another proton, its corresponding residue Asn266 in NHE1 is not involved in cation binding. Asn266 may function in maintaining the structure of the antiporter. Therefore, mutagenesis studies are needed to indentify the function of the Asp and Asn residues in controlling the Na^+^:H^+^ stoichiometry in CPA1 or CPA2 antiporters.

### PeNHX3 has a novel feature for the compensation of helix dipoles in the TM4-TM11 assembly

In NhaA, the extended chains in TM4 and TM11 helices are flanked by partial charges [38], [39]. These partial charges are stabilized by the negatively charged residue Asp133 and the positively charged residue Lys300 (Figure 7A, 7B) [38], [39]. In PeNHX3, we identified residues Tyr149 and Arg356 that were corresponding to Asp133 and Lys300 of EcNhaA, respectively (Figure 7C). Our mutagenesis analysis showed that Tyr149 and Arg356 were also functioning in compensating the partial charges of the helix dipoles in the TM4-TM11 assembly in PeNHX3 (Figure 8, 9).

Interestingly, a negatively charged residue that is equivalent to Lys300 in EcNhaA exists in all NHX antiporters with a model structure, including NHE1 (Arg425), NHA2 (Arg432) and PeNHX3(Arg356) (Figure 7; [43], [45]), suggesting that this negatively charged residue is highly conserved within NHX gene family and play essential roles in ion transport. On the other hand, the negatively charged residue corresponding to Asp133 in EcNhaA is less conserved (Figure 7; [43], [45]). So far, Asp238 in NHE1 is the only residue that has been identified to be the equivalent of Asp133 of EcNhaA [43]. PeNHX3 has a polar non-charged Tyr149 (Figure 7C), and NHA2 does not have a negatively charged residues corresponding to Asp133 [45], suggesting that there are variations at this site for charge compensation. This implies that PeNHX3 and NHA2 may have a novel mechanism for charge compensation. Indeed, in NHA2, the partial positive charge of the helix dipoles can be stabilized by the hydroxyl side chains of Ser245 and Thr462 at the N termini of the helices connecting the extended chains of TM4 and TM11, respectively [45]. Thus, more studies are needed to identify the restudies involved in charge compensation in PeNHX3.

In conclusion, the PeNHX3 structure created by homology modeling in this study sheds light on its function and catalytic mechanism. Our results demonstrate that PeNHX3 has unique features in ion binding and translocation while sharing the same structure fold and catalytic mechanism as its bacterial and human counterparts. This model structure may facilitate understanding the function and regulatory mechanism of PeNHX3 in the tree halophyte *P. euphratica* under salt stress.

## Supporting Information

Figure S1 **The pairwise alignment between PeNHX3 and EcNhaA for building the 3D structure of PeNHX3.**
(TIF)

Figure S2 **Alignment of the putative amino acid sequences of PeNHX3 and AtNHX1.**
(TIF)
