# Correction: Functional Analysis of the Na^+^,K^+^/H^+^ Antiporter PeNHX3 from the Tree Halophyte *Populus euphratica* in Yeast by Model-Guided Mutagenesis

**DOI:** 10.1371/journal.pone.0117869

**Published:** 2015-02-03

**Authors:** The PLOS ONE Staff

There is an error in the title of the third subsection of the Results. The correct title is: PeNHX3 mediates Na^+^, K^+^ and Li^+^ transport in yeast.

There is an error in the title of the first subsection of the Discussion. The correct title is: PeNHX3 may function in salt stress and Li^+^ detoxification in *P. euphratica*.

There is an error in the title of the second to last subsection of the Discussion. The correct title is: Asn187 and Asp 188 forms an ND motif that is crucial for ion binding and ion translocation in PeNHX3.
