# Supplementary figures and images for: Functional Analysis of the Na+,K+/H+ Antiporter PeNHX3 from the Tree Halophyte Populus euphratica in Yeast by Model-Guided Mutagenesis
Source: PLoS One. 2014 Aug 5;9(8):e104147. doi: 10.1371/journal.pone.0104147 (PMC4122410; doi:10.1371/journal.pone.0104147)

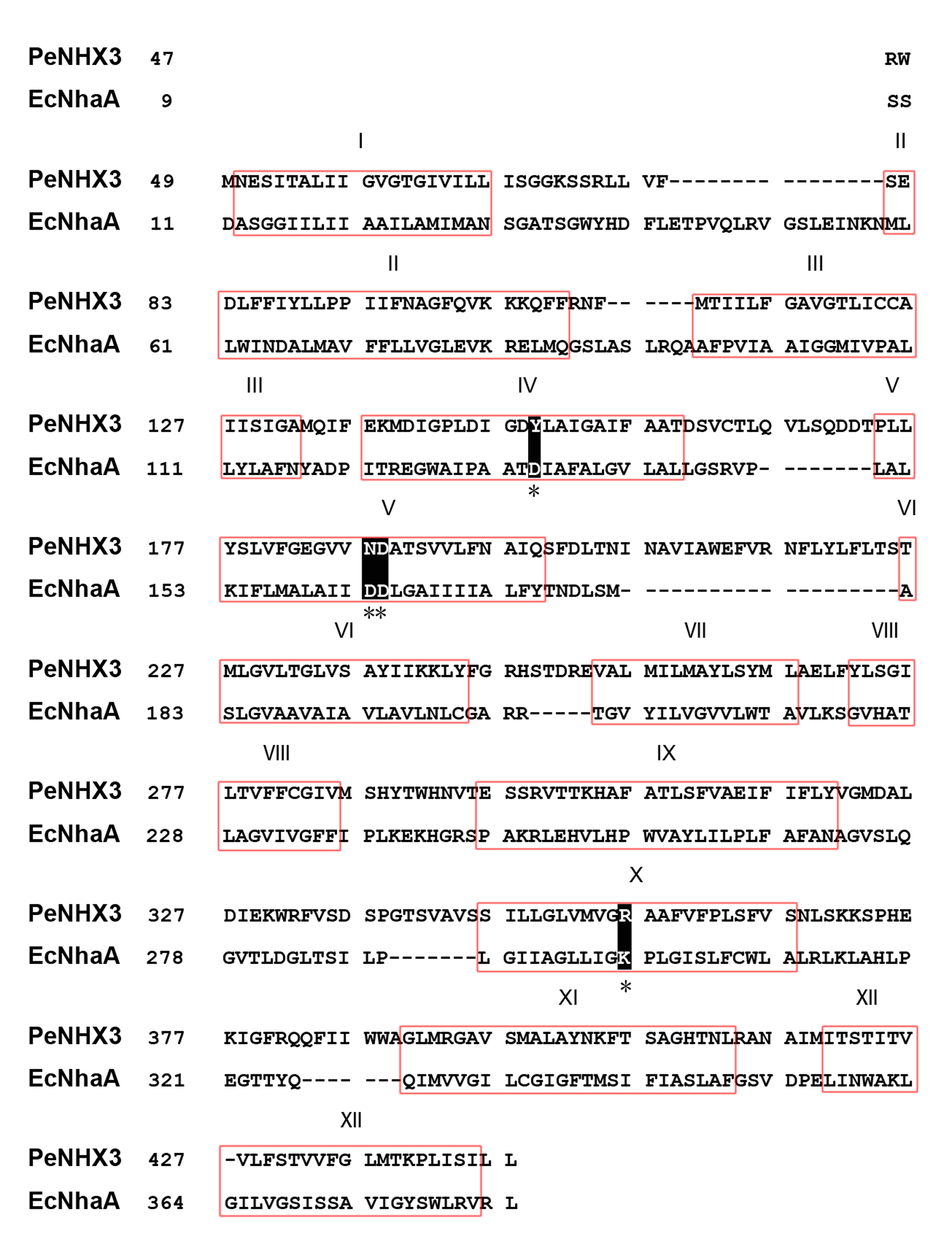

Supplement: Figure S1 — The pairwise alignment between PeNHX3 and EcNhaA for building the 3D structure of PeNHX3. (TIF) [file pone.0104147.s001.tif]

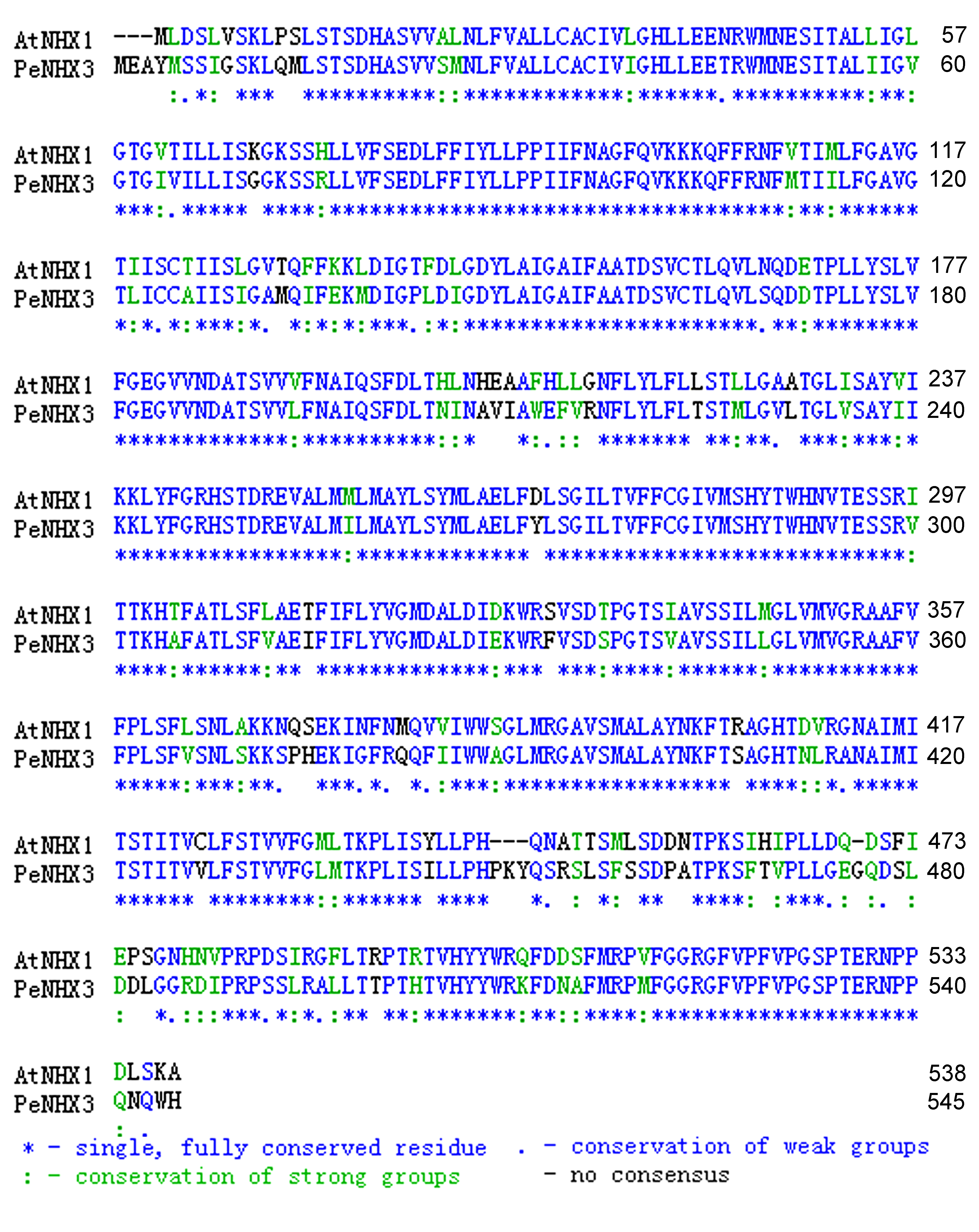

Supplement: Figure S2 — Alignment of the putative amino acid sequences of PeNHX3 and AtNHX1. (TIF) [file pone.0104147.s002.tif]
